# Dichorhaviruses Movement Protein and Nucleoprotein Form a Protein Complex That May Be Required for Virus Spread and Interacts *in vivo* With Viral Movement-Related Cilevirus Proteins

**DOI:** 10.3389/fmicb.2020.571807

**Published:** 2020-11-04

**Authors:** Mikhail Oliveira Leastro, Juliana Freitas-Astúa, Elliot Watanabe Kitajima, Vicente Pallás, Jesús Ángel Sánchez-Navarro

**Affiliations:** ^1^Unidade Laboratorial de Referência em Biologia Molecular Aplicada, Instituto Biológico, São Paulo, Brazil; ^2^Instituto de Biología Molecular y Celular de Plantas, Universidad Politécnica de Valencia-Consejo Superior de Investigaciones Científicas (CSIC), Valencia, Spain; ^3^Embrapa Mandioca e Fruticultura, Cruz das Almas, Brazil; ^4^Departamento de Fitopatologia e Nematologia, Escola Superior de Agricultura Luiz de Queiroz, Universidade de São Paulo, Piracicaba, Brazil

**Keywords:** dichorhaviruses, cileviruses, citrus leprosis pathosystem, virus movement, *in vivo* protein-protein interaction, protein membrane association and topology, mixed infection

## Abstract

*Brevipalpus*-transmitted viruses (BTVs) belong to the genera *Dichorhavirus* and *Cilevirus* and are the main causal agents of the citrus leprosis (CL) disease. In this report, we explored aspects related to the movement mechanism mediated by dichorhaviruses movement proteins (MPs) and the homologous and heterologous interactions among viral proteins related to the movement of citrus leprosis-associated viruses. The membrane-spanning property and topology analysis of the nucleocapsid (N) and MP proteins from two dichorhaviruses revealed that the MPs are proteins tightly associated with the cell membrane, exposing their N- and C-termini to the cytoplasm and the inner part of the nucleus, whereas the N proteins are not membrane-associated. Subcellular localization analysis revealed the presence of dichorhavirus MPs at the cell surface and in the nucleus, while the phosphoproteins (P) were located exclusively in the nucleus and the N proteins in both the cytoplasm and the nucleus. Co-expression analysis with the MP, P, and N proteins showed an interaction network formed between them. We highlight the MP capability to partially redistribute the previously reported N-P core complex, redirecting a portion of the N from the nucleus to the plasmodesmata at the cell periphery, which indicates not only that the MP might guide the intracellular trafficking of the viral infective complex but also that the N protein may be associated with the cell-to-cell movement mechanism of dichorhaviruses. The movement functionality of these MPs was analyzed by using three movement-defective infectious systems. Also, the MP capacity to generate tubular structures on the protoplast surface by ectopic expression was analyzed. Finally, we evaluated the *in vivo* protein–protein interaction networks between the dichorhavirus MP and/or N proteins with the heterologous cilevirus movement components, which suggest a broad spectrum of interactions, highlighting those among capsid proteins (CP), MPs, and Ns from citrus leprosis-associated viruses. These data may aid in understanding the mixed infection process naturally observed in the field caused by distinct BTVs.

## Introduction

The *Brevipalpus*-transmitted viruses (BTV) have been separated into two groups: the cytoplasmic type (BTV-C) that replicate in the cytoplasm of infected cells and are composed of viruses of genus *Cilevirus*, family *Kitaviridae* (Locali-Fabris et al., 2012; Quito-Avila et al., 2020), and the nuclear type (BTV-N), which replicate in the nucleus and are classified in the genus *Dichorhavirus*, family *Rhabdoviridae* (Dietzgen et al., 2018; Freitas-Astua et al., 2018). BTV from both genera are causal agents of citrus leprosis (CL), a re-emergent viral disease characterized by the induction of localized chlorotic and necrotic lesions on infected citrus tissues, which affects the crops from South to North America (Bastianel et al., 2010) and was recently identified in South Africa (Cook et al., 2019). Dichorhaviruses share molecular and biological characteristics with non-segmented plant rhabdoviruses that replicate in the nuclei, such as the sonchus yellow net virus (SYNV) and datura yellow vein virus (DYVV) (Dietzgen et al., 2018). In 2019, the International Committee on Virus Taxonomy (ICTV) split the former genus *Nucleorhabdovirus* into three new genera *Alphanucleorhabdovirus, Betanucleorhabdovirus*, and *Gammanucleorhabdovirus* (Freitas-Astúa et al., 2019; Dietzgen et al., 2020). This decision was based on the observation that the betanucleorhabdoviruses, like SYNV and DYVV, cluster together as sister clades with the dichorhaviruses (Freitas-Astúa et al., 2019; Dietzgen et al., 2020). Orchid fleck virus (OFV) is the type member of the genus *Dichorhavirus* and the only BTV that has a worldwide distribution. It was firstly identified in orchid plants in Japan (Kondo et al., 2003) and, in addition to Asia, occurs in the Americas (Freitas-Astúa et al., 2002), Europe (Petzold, 1971; Sauvetre et al., 2018), Oceania (Gibbs et al., 2000), and it was recently detected in the African continent (Cook et al., 2019). A citrus strain of OFV (OFV-citrus) has been found infecting several citrus genotypes in Mexico, Colombia, and South Africa causing CL symptoms (Cruz-Jaramillo et al., 2014; Roy et al., 2015b; Cook et al., 2019). More recently, citrus leprosis virus N (CiLV-N) and citrus chlorotic spot virus (CiCSV), two new dichorhaviruses also associated with CL disease, were identified in citrus plants in Brazil (Ramos-Gonzalez et al., 2017; Chabi-Jesus et al., 2018). Citrus leprosis is indeed an unusual disease, caused by viruses belonging to two completely distinct genera but sharing some similarities as the particle morphology, symptomatology in citrus plants, and the *Brevipalpus* spp. mite vector. Not only different species of viruses but also of *Brevipalpus* can be found in citrus orchards associated with CL (Sanchez-Velazquez et al., 2015; Beltran-Beltran et al., 2020). In this context, mixed infections with the cilevirus citrus leprosis virus C2 (CiLV-C2) and the dichorhavirus OFV-citrus infecting the same leaf lesion were recently identified in *Citrus sinensis* (sweet orange) in Colombia (Roy et al., 2015a).

Dichorhaviruses contain (-)ssRNA bisegmented genomes (Dietzgen et al., 2018). RNA 1 codes for the nucleocapsid protein (N), phosphoprotein (P), putative movement protein (MP), matrix protein (M), and glycoprotein (G), while RNA2 codes for the L protein, an RNA-dependent RNA polymerase (RdRp) (Kondo et al., 2014; Dietzgen et al., 2018; Freitas-Astua et al., 2018). The protein–protein interaction and subcellular map localization of the dichorhaviruses proteins in the plant cell have been explored. A recent study showed the nucleophilic properties of five coffee ring spot virus (CoRSV) proteins and their capacity to interact with each other (Ramalho et al., 2014). The MP is distributed along the cell periphery, and it is the only protein unable to interact with other CoRSV proteins. The P protein is observed exclusively into the nuclei, while the N protein is observed both in the nucleus and in the cytoplasm. The P is relocalized to the cytoplasm when co-expressed with the N, suggesting that the N could mediate the intracellular transport of infectious viral components from the nucleus to the cell periphery (Ramalho et al., 2014). In a more detailed study aiming to unravel the mechanism of OFV nuclear viroplasm formation, it was shown that N accumulates throughout the cell and P is associated with the cell nuclei, with the N being recruited to the nuclei by the presence of P, forming a intranuclear viroplasm-like structure mediated by P nuclear localization signal (NLS) (Kondo et al., 2013). In contrast, for SYNV, the genetically related betanucleorhabdovirus, both N and P proteins are separately imported into the nucleus via NLS-dependent and -independent pathway systems, respectively (Goodin et al., 2001; Deng et al., 2007; Kondo et al., 2013). Although the nuclear importing activity for dichorhavirus nucleocapsids is relatively well resolved, the mechanism as to how the core ribonucleoprotein (RNP) complex is exported from the nucleus to the plasmodesmata for cell-to-cell movement, with special emphasis on MP’s participation, still needs elucidation.

To establish systemic infection in plants, viruses have evolved genes that encode specialized proteins essential for their transport. These MPs act in synchronization with viral and host factors resulting in sophisticated movement strategies in order to ensure effective viral spread. The MPs could facilitate the intracellular virus spread from the viral sites of replication to cell periphery, then to neighboring cells through structural changes in the plant intracellular connections, the plasmodesmata (PD). Once the MP reaches the PD connection, this protein may assist the virus transport to neighboring cells in different ways: (i) as a viral vRNP complex, (ii) as a viral replication complex (VRC), (iii) as virions through a tubule-mediated mechanism, or (iv) by an intermediate mechanism, where tubular structures promote the movement of ribonucleoprotein complexes which include the coat protein rather than the entire particle (Sanchez-Navarro and Bol, 2001; Kawakami et al., 2004; Sanchez-Navarro et al., 2006; Ritzenthaler and Hofmann, 2007; Hofmann et al., 2009; Sambade and Heinlein, 2009; Navarro et al., 2019). Furthermore, some MPs might require their cognate nucleocapsid proteins in some steps of the movement process to allow viral transport (Nagano et al., 1997; Sanchez-Navarro and Bol, 2001; Sanchez-Navarro et al., 2006; Aparicio et al., 2010).

As reviewed by Leastro et al. (2015), interactions among viruses seem to be a common feature in nature. Mixed infection under natural conditions between viral species of the same or related genera may provide greater genetic diversity favoring virus infection, genetic reassortment and recombination (Brown et al., 2002; Idris and Brown, 2004; Idris et al., 2008; Leastro et al., 2015). A more extensive study on the interaction among viral proteins of members of the same or between related genera associated with the CL disease may shed light on the nature of mixed plant infection and mite colonization by both BVT-N and BTV-C. In this scenario, we selected to study the dichorhavirus MP, N, and P, and the cilevirus p32 (MP) and p29 (capsid) proteins, which are putatively associated with their movement mechanisms (Leastro et al., unpublished). Therefore, they have potential to be critical components of compatibility in the natural mixed infection process observed in the CL pathosystem. For that, we first evaluated the interaction complex formed among MP, N, and P of the dichorhaviruses CiLV-N and OFV-citrus, focusing on the mechanism of action of the MP to ensure viral movement. Then, we explored the association between dichorhavirus movement components with the key viral components associated with the movement of cileviruses, so a comprehensive discussion was generated in order to better understand the process of mixed infection between members of viral species of two distinct genera.

## Materials and Methods

### DNA Manipulation

*MP*, *N*, and *P* genes of CiLV-N (isolate Ibi1 GenBank access: KX982176.1) and OFV-citrus (isolate M2345 GenBank access: KF209275.2) were obtained from total RNA extracted from infected citrus samples. RT-PCR was performed following the manufacturer’s specifications (Thermo Fisher Scientific, United States). The cDNA was generated with specific primers for each gene, followed by PCR amplification with the primers carrying the sites *Nco*I/*Nhe*I (*N* CiLV-N), *Bsp*HI/*Nhe*I (*P* CiLV-N and *MP* OFV-citrus), *Pci*I/*Nhe*I (*MP* CiLV-N and *P* OFV-citrus), and *Nco*I/*Xba*I (*N* OFV-citrus).

For the analysis of cell-to-cell spread, a modified infectious alfalfa mosaic virus (AMV) cDNA 3 clone (pGFP/A255/CP) (Sanchez-Navarro et al., 2001), which expresses the green fluorescent protein (GFP), was used to exchange the 255 amino acids (aa) of the AMV *MP* gene with the corresponding putative *MP, N*, and *P* of CiLV-N or OFV-citrus. The genes were cloned with the compatible sites *Nco*I/*Nhe*I. The resultant constructs carrying the heterologous indicated proteins were fused with the C-terminal 44 aa (A44) of the AMV *MP*, which favors a better efficiency in movement, given that this region is responsible for the interaction to the cognate AMV capsid protein (CP) (Sanchez-Navarro et al., 2006; Aparicio et al., 2010). In addition, the *MP, N* and *P* genes containing stop codon sequences were introduced into the AMV cDNA 3 clone to generate proteins not fused with A44.

For turnip crinkle virus (TCV) assay of *in trans* movement complementation, the dichorhavirus *MPs* carrying stop codons were introduced in the expression cassette of the plasmid pSK35S-MP_TSWV_:HA-PoPit by replacing the TSWV *NSm* gene (Leastro et al., 2017a). The expression cassettes containing CiLV-N or OFV-citrus *MP* were subcloned into the pMOG_800_ binary vector by using the restriction site *Hin*dIII.

To evaluate the subcellular localization and redistribution of the proteins, the enhanced GFP (eGFP) gene was fused at the C- or N-termini of the different viral genes. For C-terminal fusion, the genes were cloned into the vector pSK35S-GFP:eGFP-PoPit (Leastro et al., 2015) replacing the GFP gene. For the protein re-localization assay, the CiLV-N and OFV-citrus *P* genes were fused at their C-termini with RFP (red fluorescent protein) using the plasmid pSK35S-GFP:RFP-PoPit. The resultant clones contained the corresponding dichorhaviruses genes fused to the eGFP or RFP, or non-fused, under the control of 35S constitutive promoter from cauliflower mosaic virus (CaMV) and the terminator from the potato proteinase inhibitor (PoPit) (Leastro et al., 2015). The correspondent expression cassettes were subcloned into the pMOG_800_ binary vector by using the restriction sites *Eco*RI/*Xho*I or *Hin*dIII. For the N termini fusions, the genes were inserted into the vector pSK 35S-eGFP:GFP-PoPit replacing the GFP and subcloned into the pMOG_800_ binary vector as above mentioned.

For the membrane association or tubule formation assays, the *MP*, *N*, and *P* dichorhaviruses proteins were human influenza hemagglutinin (HA)-tagged at their C-termini by insertion into the pSK35S-*MP*_CiLV–C_:HA-PoPit construct (Leastro et al., 2018), replacing the citrus leprosis virus C (CiLV-C) *MP* gene. Then, the expression cassettes were subcloned into the pMOG_800_ binary vector by using the restriction site *Hin*dIII.

For the BiFC analyses, membrane topology, and *in vivo* viral protein-protein interaction, we inserted the viral genes in the constructs pSK35S-NYFP:eGFP-PoPit, pSK35S-CYFP:eGFP-PoPit, pSK35S-eGFP:NYFP-PoPit, and pSK35S-eGFP:CYFP-PoPit, which permitted N-and C-terminal fusion of the enhanced yellow fluorescent protein (EYFP) fragments at the N- or C-termini of a specific assayed protein by exchange of the eGFP gene using the *Nco*I/*Nhe*I restriction sites. Detailed procedures for obtaining these plasmids were previously described (Leastro et al., 2015). In short, we fused the N-terminal 154 amino acids of the YFP (NYFP) to the N- and C-termini of the *MP*, *N*, and *P* dichorhaviruses proteins. The C-terminal 84 amino acids of the YFP (CYFP) were also fused to the N- or C-termini of the mentioned proteins. The cassettes containing the corresponding genes were subcloned into the pMOG_800_ binary vector as aforementioned.

The constructs, which contained the N- and C-termini fragments of the EYFP addressed to the cytosol (NYFPcyt and CYFPcyt) or ER (NYFPer and CYFPer) used here for BiFC topology, were obtained from Aparicio et al. (2006) and Zamyatnin et al. (2006), respectively.

The BiFC, HA-tagged and non-fused constructs carrying the *p29* and/or *MP* genes of the cileviruses CiLV-C and CiLV-C2 were obtained from Leastro et al. (2018) and Leastro et al. (submitted). All constructs and DNA manipulation steps were confirmed by plasmid DNA sequencing.

### Subcellular Fractionation, Chemical Treatment, and Western Blot Analyses

*Agrobacterium tumefaciens* strain C58 cultures were transformed with binary pMOG_800_ plasmid, harboring the dichorhavirus *MP* and *N* genes and leader peptidase (*Lep*) gene fused at their C-termini to the HA epitope. The *Lep* construct was obtained from Peiro et al. (2014b). The pUC35s-GFP-HDEL plasmid that expresses free GFP with ER retention signal was introduced by electroporation into the Agrobacterium strain GV3101. The cultures (OD_600_ 0.4) were individually agroinfiltrated in *Nicotiana benthamiana* leaves as described previously (Leastro et al., 2015). After 3 days post infiltration (dpi), the leaves were processed to obtain enriched membranous fraction. As previously related by Leastro et al. (2018), approximately 1.5 g of infiltrated leaves were ground in lysis buffer [20 mM HEPES, pH 6.8; 150 mM potassium acetate; 250 mM mannitol; 1 mM MgCl_2_ and 50 μL of protease inhibitor cocktail for plant cell (Sigma-Aldrich, Steinheim, Germany)]. The homogenate was clarified by centrifugation at 3,000 × *g* for 10 min at 4°C. The collected supernatant was ultracentrifuged at 40,000 × *g* for 40 min at 4°C to yield the soluble (S30) and the crude (P30) microsomal fractions. Microsomal pellets were resuspended in lysis buffer and divided into five fractions, to which the same volume of original lysis buffer (control aliquot); 100 mM Na_2_CO_3_ (pH 11.0); and 2M, 4M, and 8M urea were added and incubated for 30 min on ice. The supernatant fractions (S30) were collected by ultracentrifugation at 40,000 × *g* for 40 min at 4°C and the respective pellets (P30) were resuspended in the same volume with lysis buffer. Treatment of the pellet (P30) with Triton X-114 was performed separately. As referred by Kang et al. (2015), the P30 fraction was resuspended in the lysis buffer containing 1% Triton X-114 followed by incubation on ice for 30 min. Mixture was clarified by centrifugation at 10,000 × *g* for 20 min at 4°C, then the supernatant was incubated at 37°C for 10 min at room temperature to form the aqueous (AP) and the hydrophobic phase (OP), and centrifuged at 10,000 × g for 10 min at room temperature for phase separation. Finally, the OP was resuspended in lysis buffer with the same volume obtained in the aqueous phase. All the fractions were analyzed by Western blot in 12% SDS-PAGE gels. The gels were electrotransferred to polyvinylidene difluoride membranes following the manufacturer’s instructions (Amersham^TM^ Protan^®^, GE Healthcare, United States). The detection of the proteins tagged with HA or the GFP and percentage values of relative concentration of the protein gel bands was performed as described previously (Leastro et al., 2015).

### Intracellular Protein Sublocalization and Redistribution

To visualize the intracellular localization of dichorhavirus MP, N, and P proteins in plant cells, *A. tumefaciens* cultures containing the viral genes fused to the eGFP, RFP, or HA tag in pMOG_800_ binary vector were infiltrated in *N. benthamiana* leaves (OD_600_ 0.4) as described before. The plants were kept at 23°C in the light for 16 h and at 18°C in the dark for 8 h with 70% relative humidity. The fluorescence was observed between 48 and 72 h post-infiltration.

To investigate the colocalization of the dichorhaviruses proteins with the nucleus or the redistribution of the proteins, simultaneous expression of two or three proteins in individual bacteria cultures containing the correspondent binary vectors carrying the dichorhavirus genes or the organelle markers was performed. For all subcellular expression analyses, three independent experiments were performed, each one included the infiltration of three leaves per construct such as performed by Leastro et al. (2018). All images displayed are representative of at least three independent experiments.

### Organelle Markers

For the nucleus subcellular colocalization, the proteins were co-infiltrated with cultures (OD_600_ 0.1) expressing the nuclear localization signal of SV40 large T antigen fused to the RFP, kindly provided by Dr. José Navarro IBMCP, Valencia, Spain.

As described by Leastro et al. (2018) for callose staining, *N. benthamiana* leaves were infiltrated with aniline blue (Merck KGaA, Darmstadt, Germany) solution at 0.005% concentration in sodium phosphate buffer, 70 mM, pH 9.0. The leaves were infiltrated and kept in a dark room for 2 h before confocal visualization.

### Bimolecular Fluorescence Complementation Assays

All procedures for the BiFC assays were conducted as previously reported by Leastro et al. (2018). In short, *A. tumefaciens* (strain C58) cultures (OD_600_ 0.4) transformed with the corresponding binary plasmid pMOG_800_ were used to infiltrate *N. benthamiana* plants as previously mentioned. At 4 dpi, the fluorescence reconstitution was observed. To increase the expression, in order to allow a better visualization of the fluorescence signal, all protein pair combinations were co-expressed with the silencing suppressor HCPro from tobacco etch virus.

For the BiFC assay aiming to characterize the topology of N and MP dichorhavirus proteins, the N and MP carrying the NYFP or CYFP fused at their N- or C-termini were transiently expressed with the counterpart addressed to the cytosol (N-YFPcyt and C-YFPcyt) or ER lumen (C-YFRer and N-YFPer) (Leastro et al., 2018). All protein pair combinations performed in the BiFC topology analyses are shown in Table 1.

**TABLE 1:** Protein pair combinations performed in the BiFC topology assay for MPs and Ns proteins of the CiLV-N and OFV-citrus.

Membrane topology		NYFP-Cyt	CYFP-Cyt	NYFP-ER	CYFP-ER
Control	NYFP-ER		−		+
	NYFP-Cyt		+		−

CiLV-N	N-NYFP		+		−
	N-CtYFP	−		−	
	NYFP-N		+		−
	CYFP-N	−		−	
	MP-NYFP		+		−
	MP-CYFP	−		−	
	NYFP-MP		+		−
	CYFP-MP	−		−	

OFV-citrus	N-NYFP		+		−
	N-CYFP	−		−	
	NYFP-N		+		−
	CYFP-N	−		−	
	MP-NYFP		+		−
	MP-CYFP	−		−	
	NYFP-MP		+		−
	CYFP-MP	−		−	

*The symbols − and + correspond to the absence and presence of fluorescence signals, respectively. NYFP and CYFP correspond to half N- and C-terminal of the YFP protein, respectively.*

For *in vivo* protein-protein interaction, the dichorhaviruses (N, P, and MP) and cileviruses (p29 and MP) proteins were fused at their N- and C- termini with NYFP and CYFP. In dimerization analysis, performed individually for each dichorhavirus or cilevirus protein, the indicated pair of proteins was transiently expressed in *N. benthamiana* as described previously by Leastro et al. (2018). The same was performed in the heterologous associations; however, the combination pairs were performed between different proteins from the same species (intra-association) or different species of the same genus (inter-association), or also between species of different genera (inter-association between genera). Tables 2, 3 summarize all combinations performed in dimerization and heterodimerization assays, respectively. For BiFC analyses, three independent experiments were performed, each one included the infiltration of three leaves per construct. All BiFC images displayed are representative of at least three independent experiments.

**TABLE 2:** Protein pair combinations performed in the BiFC dimerization assay.

Dimers	CiLV-N			OFV-citrus		
	N	MP	P	N	MP	P
ORF-NYFP + ORF-CYFP	−	−	+	+	+	+
ORF-NYFP + CYFP-ORF	+	−	+	+	−	+
NYFP-ORF + ORF-CYFP	−	+	+	−	−	+
NYFP-ORF + CYFP-ORF	+	+	+	+	−	+
Negative controls						
CYFP-ORF + NYFP-Cyt	−	−	−	−	−	−
ORF-CYFP + NYFP-Cyt	−	−	−	−	−	−
NYFP-ORF + CYFP-ER	−	−	−	−	−	−

*The symbols − and + correspond to the absence and presence of fluorescence signals, respectively. NYFP and CYFP correspond to half N- and C-terminal of the YFP protein, respectively.*

**TABLE 3:** Protein pair combinations performed in the BiFC heterologous assays.

Heterologous assays	p29/MP CiLV-C2	N/MP CiLV-N	P/N CiLV-N	P/MP CiLV-N	N/MP OFV
ORF-NYFP + ORF-CYFP	+	+	−	+	−
ORF-NYFP + CYFP-ORF	−	−	+	+	−
NYFP-ORF + ORF-CYFP	−	−	−	+	−
NYFP-ORF + CYFP-ORF	−	−	+	+	+
ORF-CYFP + ORF-NYFP	+	−	+	+	−
ORF-CYFP + NYFP-ORF	−	−	+	+	−
CYFP-ORF + ORF-NYFP	+	−	+	+	−
CYFP-ORF + NYFP-ORF	−	+	+	+	−
Negative controls					
CYFP-ORF + NYFP-cyt	−	−	−	−	−
ORF-CYFP + NYFP-cyt	−	−	−	−	−
NYFP-ORF + CYFP-ER	−	−	−	−	−

	**P/N OFV**	**P/MP OFV**	**p29 CiLV-C/p29 CiLV-C2**	**MP CiLV-C/MP CiLV-C2**	**p29 CiLV-C/MP CiLV-C2**

ORF-NYFP + ORF-CYFP	−	+	+	−	−
ORF-NYFP + CYFP-ORF	+	+	+	−	−
NYFP-ORF + ORF-CYFP	+	−	+	−	−
NYFP-ORF + CYFP-ORF	+	+	+	−	−
ORF-CYFP + ORF-NYFP	+	+	+	−	+
ORF-CYFP + NYFP-ORF	+	+	+	−	−
CYFP-ORF + ORF-NYFP	+	+	+	−	+
CYFP-ORF + NYFP-ORF	+	+	+	−	−
Negative controls					
CYFP-ORF + NYFP-cyt	−	−	−	−	−
ORF-CYFP + NYFP-cyt	−	−	−	−	−
NYFP-ORF + CYFP-ER	−	−	−	−	−

	**p29 CiLV-C2/MP CiLV-C**	**N CiLV-N/N OFV**	**MP CiLV-N/MP OFV**	**N CiLV-N/MP OFV**	**N OFV/MP CiLV-N**

ORF-NYFP + ORF-CYFP	−	+	−	−	−
ORF-NYFP + CYFP-ORF	+	+	−	−	+
NYFP-ORF + ORF-CYFP	−	−	+	−	−
NYFP-ORF + CYFP-ORF	−	+	−	−	−
ORF-CYFP + ORF-NYFP	+	−	+	−	−
ORF-CYFP + NYFP-ORF	+	−	+	−	−
CYFP-ORF + ORF-NYFP	+	+	+	−	−
CYFP-ORF + NYFP-ORF	−	−	+	−	+
Negative controls					
CYFP-ORF + NYFP-cyt	−	−	−	−	−
ORF-CYFP + NYFP-cyt	−	−	−	−	−
NYFP-ORF + CYFP-ER	−	−	−	−	−

					**Intra-association control**

	**MP CiLV-C2/MP OFV**	**p29 CiLV-C2/N OFV**	**p29 CiLV-C2/MP OFV**	**MP CiLV-C2/N OFV**	**MP CiLV-C/p24 CiLV-C**

ORF-NYFP + ORF-CYFP	+	+	−	−	−
ORF-NYFP + CYFP-ORF	−	+	−	−	−
NYFP-ORF + ORF-CYFP	+	−	−	−	−
NYFP-ORF + CYFP-ORF	−	−	+	−	−
ORF-CYFP + ORF-NYFP	−	+	+	−	−
ORF-CYFP + NYFP-ORF	−	+	+	−	−
CYFP-ORF + ORF-NYFP	+	+	+	−	−
CYFP-ORF + NYFP-ORF	−	+	−	−	−
Negative controls					
CYFP-ORF + NYFP-cyt	−	−	−	−	−
ORF-CYFP + NYFP-cyt	−	−	−	−	−
NYFP-ORF + CYFP-ER	−	−	−	−	−

	**Inter-association controls**				

	**CP AMV/MP CiLV-C**	**N TSWV/MP CiLV-C**			

ORF-NYFP + ORF-CYFP	−	−			
ORF-NYFP + CYFP-ORF	−	−			
NYFP-ORF + ORF-CYFP	−	−			
NYFP-ORF + CYFP-ORF	−	−			
ORF-CYFP + ORF-NYFP	n.d	−			
ORF-CYFP + NYFP-ORF	n.d	−			
CYFP-ORF + ORF-NYFP	n.d	−			
CYFP-ORF + NYFP-ORF	n.d	−			
Negative controls					
CYFP-ORF + NYFP-cyt	−	−			
ORF-CYFP + NYFP-cyt	−	−			
NYFP-ORF + CYFP-ER	−	−			

*The symbols − and + correspond to the absence and presence of fluorescence signals, respectively. NYFP and CYFP correspond to half N- and C-terminal of the YFP protein, respectively. OFV = OFV-citrus. n.d = non-determined. Additional negative controls are presented for intra- and inter-association assays.*

### Confocal Laser Scanning Microscopy

Fluorescence images of the leaf discs from *N. benthamiana* were captured with the aid of a confocal laser scanning microscope Zeiss LSM 780 model. Aniline blue fluorochrome was excited at 405 nm, and emission was captured at 410–480 nm. GFP fusion proteins were excited at 488 nm and emission was captured at 495–520 nm. YFP was excited at 514 nm and emission was captured at 520–560 nm. The mRFP fluorophore was excited at 552 nm and emission was captured at 585–610 nm. The images were prepared using Fiji ImageJ program (version 2.0r).

### Protoplast Preparation

*N. benthamiana* leaves (three per each construct) were individually infiltrated with *A. tumefaciens* (strain C58) cultures (OD_600_ 0.5) transformed with the corresponding binary vector pMOG_800_ containing the dichorhavirus *MPs* or co-infiltrated in combination with the *N*-HA, *P*-HA, and *N*-HA + *P*-HA. The infiltrated leaves were used for protoplasts isolation (Loesch-Fries et al., 1985). “Each image-frame expressing GFP represents the visualization of several protoplasts (about 15–20) per assay for each protein combination analyzed” (Leastro et al., 2017b). GFP expression in protoplast was analyzed with a Zeiss LSM 780 confocal laser-scanning microscope.

### Alfalfa Mosaic Virus Assay

For analyses of cell-to-cell movement, the cassette from plasmids containing all proteins assayed inserted into AMV 3 cDNA, was amplified with specific primer pairs, and the generated amplicons were used directly as templates for *in vitro* transcription with T7 RNA polymerase (Takara Bio Inc., United States). For *MP*, *N*, and *P* co-inoculation, we balanced the concentration of AMV RNA3 transcripts carrying the heterologous genes. The quantification was performed with agarose gel electrophoresis using an RNA ladder (RiboRuler High Range RNA Ladder, Thermo Scientific) and several dilutions of the transcribed RNAs. Next, transgenic *N. tabacum* P12 plants that express the polymerase proteins P1 and P2 of AMV (van Dun et al., 1988) were grown and inoculated with RNA transcripts, as previously described (Taschner et al., 1991). The chimeric AMV RNA 3 expressing the AMV MP wild type (wt) was obtained from Leastro et al. (2017b). As reviewed by Leastro et al. (2020), three independent experiments were performed, each one included the infiltration of three leaves of P12 plants per construct. The foci images in P12 plants were taken with the aid of a Leica MZ16F fluorescence stereomicroscope.

### Turnip Crinkle Virus Complementation Assay

This system is based on the *in trans* complementation of the movement-deficiency phenotype of a TCV MP deletion mutant that expresses GFP (TCVΔ92-sGFP) (Powers et al., 2008). The trans-complementation assay was performed as previously described by Martinez-Perez et al. (2019). In short, three leaves of *N. benthamiana* per plant were infiltrated with *A. tumefaciens* cultures containing the empty pMOG_800_ or expressing the LEP protein (negative controls), *MP* of CiLV-C (positive control) (both obtained from Leastro et al., 2020) or the pMOG_800_ constructs expressing the CiLV-N or OFV-citrus *MPs* at OD_600_ 1. Next, the pTCVΔ92-sGFP plasmid was linearized with *Xba*I and transcripts were inoculated at 1 day post infiltration. TCVΔ92-sGFP infectious RNA transcripts were mechanically inoculated onto the abaxial surfaces of the infiltrated leaves. Cell-to-cell movement was evaluated at 3 days after inoculation, with a Leica MZ16F fluorescence stereomicroscope. Each assay was repeated three times.

### Tobacco Mosaic Virus Complementation Assay

The pDsRedTMV-wt plasmid (provided by Dr. S. Chapman, Scottish Crop Research Institute) (Canto and Palukaitis, 2002) carrying a tobacco mosaic virus (TMV) infectious clone expressing the DsRed fluorescent protein, was modified to eliminate the *MP* gene (pDsRedTMV-ΔMP). The new TMV construct was linearized with *Kpn*I and transcribed with T7 RNA polymerase. The infectious TMV transcripts were inoculated onto *N. benthamiana* leaves, previously infiltrated (1 day) with *A. tumefaciens* cultures containing the pMOG_800_ constructs expressing the LEP protein (negative control), *MP* of CiLV-C (positive control), or the CiLV-N or OFV-citrus *MPs* at an OD_600_ 1, as described above. The fluorescent signal was monitored at 3 days post inoculation with a Leica MZ16F fluorescence stereomicroscope.

## Results

### Dichorhavirus MPs Are Membrane Proteins, While Ns Are Not Membrane-Associated

**FIGURE 1:**
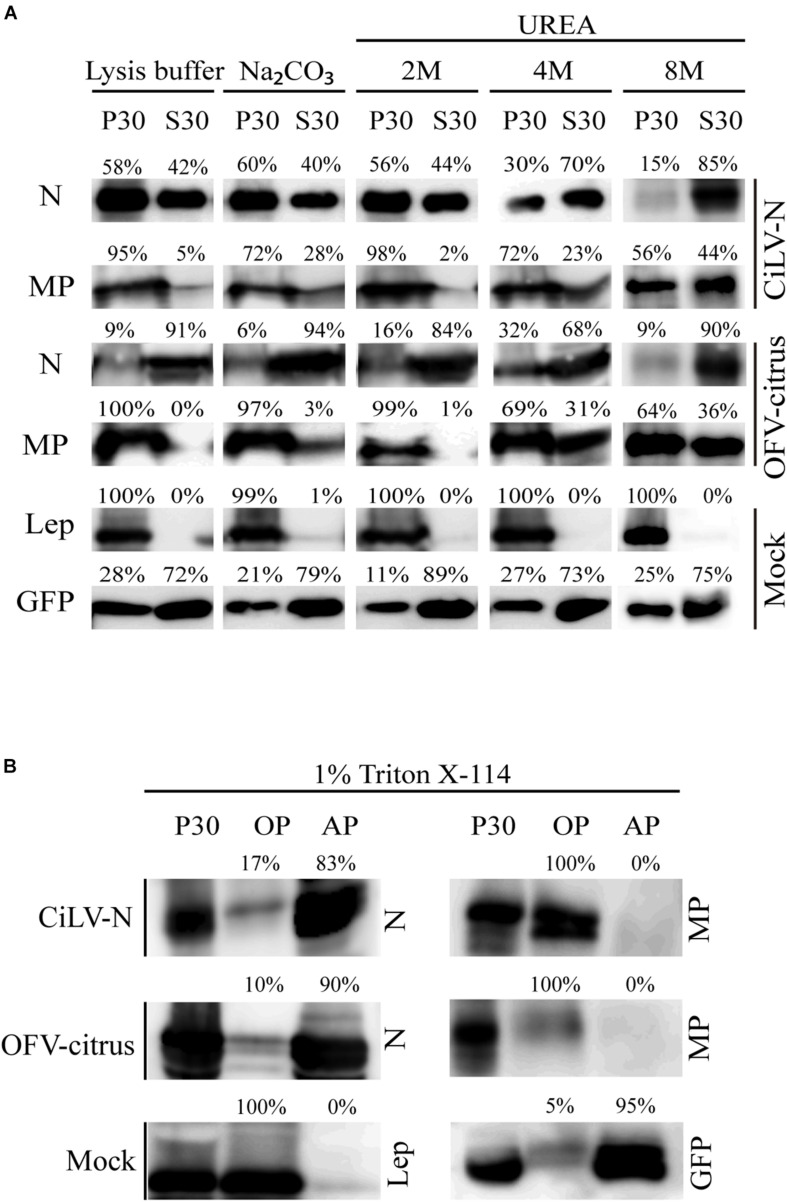
Membrane association analysis of dichorhavirus MP and N proteins. **(A)** Separation into membranous and soluble fraction of CiLV-N and OFV-citrus MP and N proteins expressed *in planta*. Respective proteins targeted with HA were expressed in *Nicotiana benthamiana* leaves by agroinfiltration. As controls, we used leaf protein extracts containing unfused expressed eGFP (non-membrane) and the HA-tagged Lep (leader peptidase) (integral membrane) proteins, respectively. The supernatant from ultracentrifugation after membrane fractioning (S30), and comparable pellet (P30), untreated and alkaline or urea (2M, 4M, and 8M) treatments were analyzed by Western blot method using an anti-NtGFP antibody (Sigma-Aldrich, Steinheim, Germany) or anti-HA antibody (Thermo Fisher Scientific, Waltham, MA, United States). Relative quantification values are presented. **(B)** Triton X-114 partitioning assay of CiLV-N and OFV-citrus MPs and Ns. The P30 fractions subjected to treatment with Triton X-114 were separated in aqueous (AP) and organic (OP) phases. Equivalent amounts of fractions were analyzed by Western blot, and the same controls mentioned above were used.

Association between movement proteins and host membranes seems to be an essential factor for virus transport, being a feature constantly identified in this class of viral proteins (Peremyslov et al., 2004; Martínez-Gil et al., 2009; Peiro et al., 2014b; Leastro et al., 2015; Pitzalis and Heinlein, 2017; Leastro et al., 2018). Thus, in order to analyze membrane association of dichorhavirus MP and N, we prepared subcellular microsomal fraction from *N. benthamiana* leaves transiently expressing the CiLV-N N, CiLV-N MP, OFV-citrus N, and OFV-citrus MP proteins fused to the HA epitope. As controls, we used leaf protein extracts containing transiently expressed free eGFP and the HA-tagged Lep protein, which are, respectively, non-membrane and integral membrane proteins (Peiro et al., 2014b; Leastro et al., 2018). High-speed ultracentrifugation was performed to separate the plant leaf lysed extract, containing the above mentioned proteins, into pellet (P30) and supernatant (S30) fractions. To identify the type of interaction, we first washed the membrane-rich fraction from each sample with Na_2_CO_3_, to release soluble luminal proteins from microsomes (Peremyslov et al., 2004; Leastro et al., 2015, 2018). With this treatment, most of the CiLV-N and OFV-citrus MPs remained associated with the membranous fraction (Figure 1A, P30 72% for CiLV-N MP and 97% for OFV-citrus MP), suggesting that these proteins are tightly associated with membranes. As expected, the integrated membrane Lep protein control also remained associated with the membranous fraction (Figure 1A, P30 99%). In agreement with the non-membrane associated GFP control, a considerable portion of the N proteins remained in the soluble fraction (Figure 1A, S30 40% for CiLV-N N, 94% for OFV-citrus N and 79% for GFP), suggesting that these proteins are not associated with host membrane. After the sequential 2M, 4M, and 8M urea treatment, all polypeptides bound to membranes should be released, except for the integral membrane proteins (Martínez-Gil et al., 2009; Leastro et al., 2015, 2018). The HA-tagged MPs were also detected in the soluble fraction (Figure 1A, 8M 44% for CiLV-N MP and 36% for OFV-citrus MP), in contrast with the Lep control that remained in the pellet fraction (Figure 1A, 8M 100%). This result suggests that dichorhavirus MP might be a peripheral protein tightly associated to membrane.

To further investigate the membrane association of the MPs and N proteins, a Triton X-114 partitioning assay was performed. In this treatment, two aqueous (AP) and organic phases (OP) are formed, in which integral membrane proteins should be portioned into the OP, while the AP should contain solube and non-integral membrane proteins (Bordier, 1981). When the P30 fractions were treated with Triton X-114, the dichorhavirus MPs were detected mostly in the OP, whereas the N proteins were associated to the AP (Figure 1B). As expected, the Lep and GFP control proteins were recovered from OP and AP, respectively (Figure 1B). Collectively, these findings indicate that the membrane insertion capability of the MPs lies between an integral membrane protein and a peripheral membrane protein, where probably these MPs do not span the lipid bilayer. For the N proteins, they behave as non-membrane associated proteins.

### The Termini of the Dichorhavirus MPs Are Oriented Toward the Cytoplasmic Face of the Biological Membranes

**FIGURE 2:**
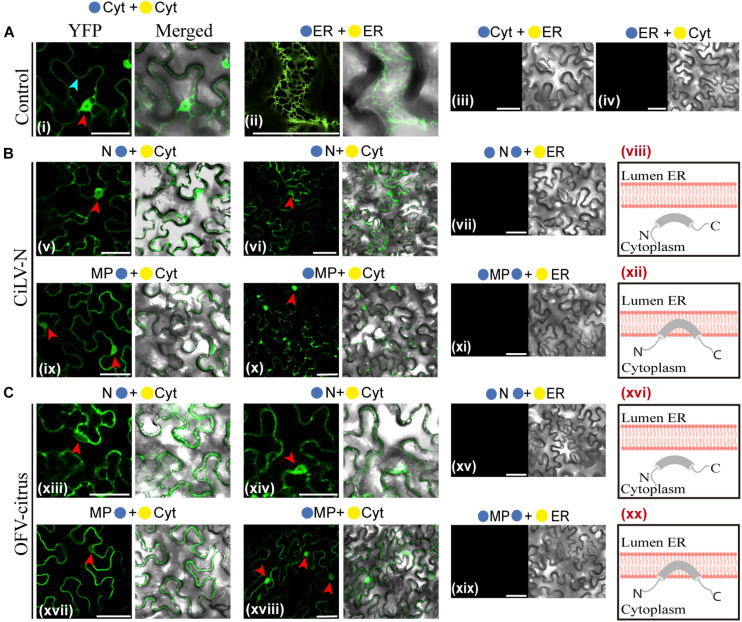
Dichorhavirus MPs are associated to membrane with their termini exposed to the cytosol. Subcellular localization (cytosolic face or ER lumen) of the N- or C-termini of the CiLV-N N, MP **(B)** and OFV-citrus N, MP **(C)**. All proteins carrying the N-terminal (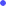) or C-terminal (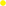) YFP fragments fused at their N-(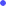/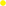-ORFs) or C-termini (ORFs-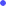/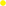) were transiently co-expressed in *N. benthamiana* leaves with corresponding complementary YFP fragment addressed to the cytosol face (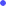-cyt or 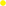-cyt) or the lumen of the ER (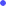-ER or 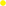-ER). Images reveal the topology of the C-termini (v,ix,xiii,xvii) or N-termini (vi, x, xiv, and xviii) of the respective dichorhavirus proteins. Red and blue arrows indicate the cell nucleus and cytoplasm, respectively. Positive and negative controls are presented in **(A)**. Hypothetical topologic models are presented at the panels to the right of the figure for each respective protein (viii,xii, xvi,xx). All images contain two pictures corresponding to the YFP signal or merged with bright field. The fluorescence was monitored at 4 days post-infiltration using a confocal Zeiss LSM 780 model. Bars correspond to 50 μm.

BiFC assays were performed to determine the subcellular compartments in which the N- or C-termini of the dichorhavirus MPs are exposed. As additional controls, we also tested the N proteins, although they are not membrane-associated proteins. BiFC constructs containing the MPs or Ns of CiLV-N and OFV-citrus were co-expressed with the counterpart of EYFP targeted to the endoplasmic reticulum (ER) (C-YFPer or N-YFPer) or to the cytosol/nucleus (C-YFPcyt or N-YFPcyt). For this BiFC topological assay, all protein pair combinations are shown in Table 1. “Reconstitution of the fluorescence-competent EYFP structure indicated the *in vivo* localization of the fused/inserted YFP half in the appropriate compartment” (Leastro et al., 2018). Confocal images showed fluorescence reconstitution when the two halves of the EYFP were co-expressed in the same subcellular compartment (N-YFPcyt + C-YFPcyt or N-YFPer + C-YFPer; Figures 2Ai,ii). However, no fluorescence signal was revealed when the two EYFP were co-expressed in different compartments (N-YFPcyt + C-YFPer or N-YFPer + C-YFPcyt; Figures 2Aiii,iv). For NYFP-MPs and MPs-NYFP, the reconstitution of the fluorescence was only observed when the respective proteins were co-expressed with C-YFPcyt, indicating that MPs termini were exposed to cytosolic compartment (Figures 2Bix,x,Cxvii,xviii). Also, fluorescence signal was visualized into the nucleus (see red arrows), suggesting the capability of these MPs to access this compartment but also indicating that both termini of the proteins are exposed to the inner part of the nucleus. Similar results were observed using the non-associated membrane N proteins, in which part of the fluorescence reconstitution was observed in the cytoplasm and part into the cell nuclei (Figures 2Bv,vi,Cxiii,xiv; red arrows indicate the nuclei), indicating the presence of this protein both in the cytoplasm and in the nucleus. No fluorescent reconstitution was observed when both MP and N proteins, fused to any of the YFP fragments, were co-expressed with the counterpart of the YFP addressed to the ER (Figures 2Bvii,xi,Cxv,xix). Taken together, these results show that the N- and C-termini of the dichorhavirus MPs are oriented toward the cytoplasmic face of the biological membrane and also into the inner part of the nucleus membrane.

### Subcellular Localization of the MP, N, and P Proteins

**FIGURE 3:**
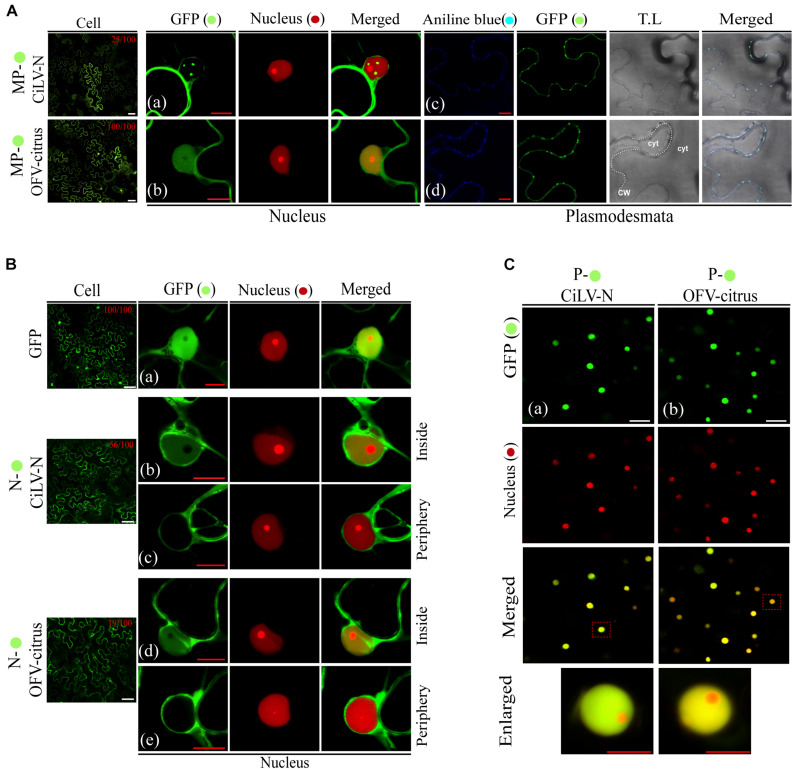
Intracellular distribution and structure formation from ectopic expression of the dichorhavirus MPs, Ns, and Ps. The CiLV-N and OFV-citrus MP, N, and P fused at their C-termini with eGFP (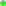) were co-expressed with mRFP(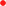) nucleus marker or aniline blue (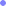) fluorochrome (plasmodesmata marker) in epidermal cells of *N. benthamiana*. Fluorescence signals were captured 48–72 h post-infiltration with confocal microscope Zeiss LSM 780 model. The green (GFP), red (mRFP) channels, and merged images are shown for each protein expressed with nucleus marker. The blue (aniline blue), green, transmitted light (T.L), and merged images are shown for MP expressed with plasmodesma marker. The dotted line in the transmitted light image indicates the cell wall (CW). Cyt = cytoplasm. The numbers at the top of cell panels correspond to the number of fluorescent cells showing fluorescence in the nucleus, i.e., 25/100 indicates that 25 out of 100 fluorescent cells showed the presence of GFP in the nuclei. The free-eGFP expression is located as a diffuse signal distributed in the cytoplasm and nucleus **(Ba)**. **(A)** Image shows the CiLV-N MP-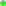 expression in punctate structures into the nuclei co-localizing with nuclei marker **(a)**, and a signal distributed at cell membrane periphery, colocalized in punctate structures with plasmodesmata along the cell periphery **(c)**. **(b)** OFV-citrus MP-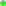 expression in a diffuse signal evenly distributed into the nucleus co-localizing with nuclei marker, and co-localizing in punctate structures with plasmodesmata along the cell periphery **(d)**. **(B)** CiLV-N and OFV-citrus N-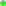 expression in a diffuse signal distributed within some cell nuclei co-localizing with nuclei marker (**b** and **d**). GFP-empty nuclei are also visualized (**c** and **e**). N-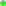 signal is also distributed throughout the cytoplasm for both viruses. **(C)** The P-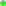 expression is visualized exclusively in cell nuclei colocalized with nuclei marker for both CiLV-N **(a)** and OFV-citrus **(b)**. Images below show in higher magnification the respective nuclei indicated in the red dashed boxes. Red and white bars correspond to 10 and 50 μm, respectively.

The OFV (orchid strain) N-P protein complex is associated in the inner part of the cell nuclei, which are thought to represent the viroplasm-like structure (Kondo et al., 2013). In order to expand the understanding about dichorhavirus transport mechanism, we evaluated the intracellular distribution of the N-P complex in presence of the cognate MP, as well as the putative interaction among the three proteins. We first characterized the intracellular localization of the MP, N, and P proteins from two different dichorhaviruses (CiLV-N and OFV-citrus). The proteins, carrying the eGFP at their N- or C-termini were transiently expressed in *N. benthamiana* leaves, alone or with a nuclear marker. The fluorescent GFP signal derived from both MPs was visualized into the nuclei and along the cell periphery. However, the intranuclear distribution of the CiLV-N MP:eGFP was organized in fluorescent punctate bodies, while the OFV-citrus MP:eGFP generated a diffuse signal (Figures 3Aa,b, respectively), similar to that observed for the free eGFP (Control, Figure 3B, cell panels), which colocalized with the nuclear marker. A quantitative analysis of 100 fluorescent cells revealed that the nuclear localization of CiLV-N MP and OFV-citrus MP was observed in 25 and 100% of the cells, respectively (Figure 3A, cell panels). Finally, the infiltration of fluorochrome aniline blue (a plasmodesmata marker staining the callose) on *N. benthamiana* leaves expressing the respective MPs, revealed that the MP-derived punctate structures observed at the cell periphery colocalize with callose deposits (Figures 3Ac,d), suggesting its association with PD. No punctate structures were observed in the negative control expressing free GFP (data not shown).

Previous results showed that individual ectopic expression of P and N proteins of OFV (orchid strain) are localized predominantly into the nucleus and the cytoplasm of *N. benthamiana* leaves, respectively (Kondo et al., 2013). The fluorescent signal derived from the transient expression of the OFV N:eGFP (citrus strain) was visualized in the cytoplasm but also in the nuclei of 19% (19 out of 100) fluorescent cells (Figure 3B, cell panel and Figures 3Bd,e). Similar fluorescent pattern was observed from the transiently expressed CiLV-N N:eGFP construct, except for the higher percentage of cells (56%; 56 out of 100) showing fluorescence into the nuclei (Figure 3B, cell panels, Figures 3Bb,c). Finally, the fluorescent signal derived from the P:eGFP proteins was predominantly located into the cell nuclei for both dichorhaviruses (Figures 3Ca,b).

For some dichorhavirus proteins, the presence of the eGFP at their N termini (i.e., eGFP-MP) revealed an impairment in the protein subcellular localization (data not shown). Accordingly, here we present the subcellular localization of constructs containing the free N-termini.

### The MP Dimerizes and Associates With the N and P Proteins, *in vivo*

**FIGURE 4:**
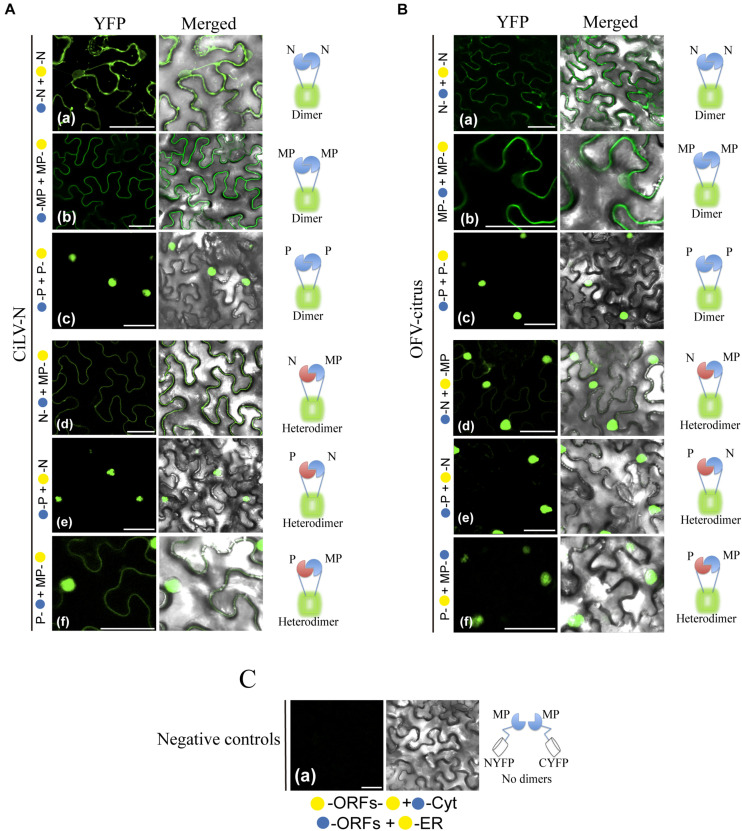
BiFC analyses suggest dimer and heterologous interactions between dichorhavirus MP, N, and P. CiLV-N and OFV-citrus Ns, Ps and MPs carrying N-terminal (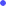) or C-terminal (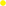) YFP fragments fused at their N- (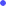/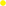
–ORFs) or C-termini (ORFs-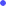/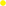) were transiently co-expressed in *N. benthamiana* leaves by agroinfiltration. Confocal microscopy images corresponding to the representative fusion protein pair combination assayed for dimeric and heterologous interactions. Representative protein pair combinations are indicated at the left of each image; all combinations performed are shown in Tables 2, 3. All images contain two pictures corresponding to the YFP signal or merged with bright field. **(Aa)** CiLV-N and **(Ba)** OFV-citrus N dimerizations with YFP signal located in the nucleus and cytoplasm. **(Ab)** CiLV-N and **(Bb)** OFV-citrus MP dimerizations with YFP signal located along the cell periphery and also visualized into the nucleus. **(Ac)** CiLV-N and **(Bc)** OFV-citrus P dimerizations with YFP signal exclusively localized into the nuclei. **(Ad)** CiLV-N N-MP intra-association with YFP signal distributed along the cell periphery, while for OFV-citrus N-MP **(Bd)** intra-association the YFP signal is more evident into the nuclei, but also located throughout cytoplasm. **(Ae)** CiLV-N and **(Be)** OFV-citrus P-N associations showing the YFP fluorescence reconstitution into the nuclei. **(Af)** CiLV-N P-MP association results in YFP signal in the nucleus and distributed along the cell periphery, while for OFV-citrus P-MP **(Bf)**, the YFP signal is exclusively into the nucleus. **(C)** Negative controls correspond to the expression of the dichorhavirus proteins in combination with Cyt or ER BiFC vectors. The left of each image has a representative scheme of the positive (dimers) or negative (no dimers) interaction corresponding to all proteins assayed. All images displayed are representative of at least three independent experiments. Bars correspond to 50 μm.

Next, BiFC analyses were performed to address the capability of interaction between the MP with the N-P complex. To do this, the MPs, Ns, and Ps of CiLV-N and OFV-citrus were fused at their N- or C- termini with the NYFP or CYFP fragments, and transient expression of different fusion protein pair combinations was performed (Table 2) by agroinfiltration of *N. benthamiana* leaves. First, we evaluated the dimer formation for the three proteins. Reconstitution of YFP fluorescence localized along the cell periphery and nucleus was visualized for the CiLV-N and OFV-citrus MPs (Figures 4Ab,Bb, respectively), suggesting the capacity of this protein to self-interact. The suggested dimer formation was also visualized for the N and P proteins of both OFV-citrus (Figures 4Aa,c) and CiLV-N (Figures 4Ba,c). The reconstituted fluorescence signal derived from the N dimerization was distributed throughout the cytoplasm and some nuclei; meanwhile, P dimerization was predominantly into the nucleus. No fluorescence signal was observed when the MPs, Ns, and Ps carrying the CYFP fragment fused at their N- or C-termini were co-expressed with the counterpart NYFP targeted to the cytosol or to the ER (negative controls, Figure 4C).

Next, we evaluated the heterologous association between the proteins. Reconstitution of the fluorescence was observed for the combinations between N-MP, P-N, and MP-P for both dichorhaviruses (Figures 4Ad–f for CiLV-N and Figures 4Bd–f for OFV-citrus). The same negative controls presented in the dimerization assay were also applied to the heterologous interactions. Table 3 summarizes all findings of the transient heterologous combination and reports all different fusion protein pair combinations performed. The fluorescence reconstitution from the interaction between the N and MP proteins was observed spread throughout the cytoplasm for CiLV-N, mostly visualized to the cell periphery (Figure 4Ad). In contrast, for OFV-citrus, it was localized more expressively into the nucleus, but also in lesser amounts in the cell periphery when compared to CiLV-N (Figure 4Bd). For both dichorhaviruses, the P-N capsid complex was localized predominantly into the nuclei (Figures 4Ae,Be). For CiLV-N, the MP-P complex was distributed into the nuclei and in the cell periphery (Figure 4Af), in contrast to the OFV-citrus, where the reconstitution of the fluorescence was visualized predominantly into the nucleus (Figure 4Bf).

These observations reveal an interaction network formed among the MP, N, and P dichorhaviruses proteins, indicating a role of the MP in recruiting N and P to the cell periphery, most evident in the analysis with CiLV-N proteins.

### Dichorhavirus MP Mediates the Relocalization of N Protein From the N-P Core Complex to the Plasmodesma Structures

**FIGURE 5:**
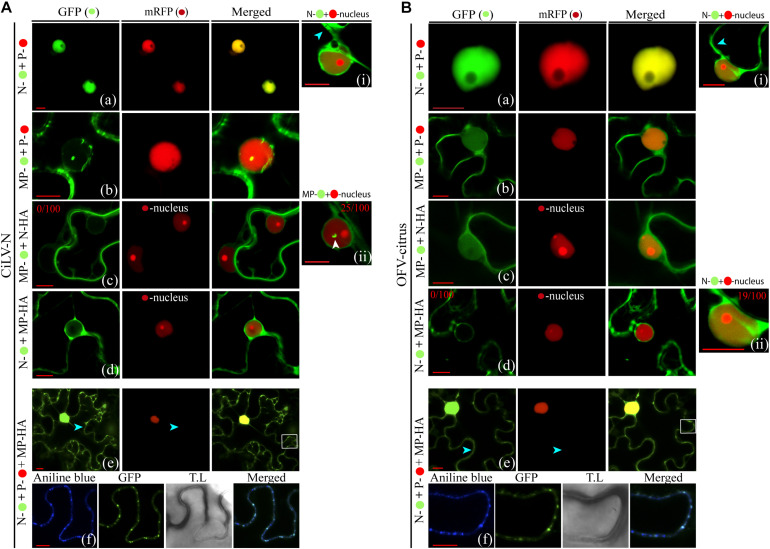
Redistribution of the N protein from the N-P complex to the cell periphery and plasmodesmata upon co-expression with the MP. Confocal imagens of CiLV-N and OFV-citrus Ns-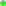, Ns-HA, Ps-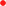, MPs-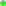 and MPs-HA transiently co-expressed in *N. benthamiana* leaves visualized at 48–72 h post-infiltration. The protein pair combinations performed among these constructions are: N-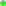 + P-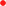 **(Aa,Ba)**, MP-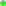 + P-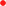 **(Ab,Bb)**, MP-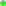 + N-HA + 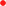-nucleus marker **(Ac,Bc)**, N-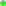 + MP-HA + 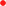-nucleus marker **(Ad,Bd)**, and N-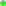 + P-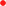 + MP-HA **(Ae,Be)**. Blue arrows indicate GFP signal in the cytoplasm and white arrows indicate the GFP signal into the nucleus. The numbers at the top of **Ai, Ac, Aii, Bd, and Bii** panels corresponds the number of nuclei visualized expressing the correspondent protein fused to GFP from 100 counted nuclei. White boxes correspond to high magnification to highlight callose deposits stained with aniline blue (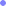) along the cell periphery **(Af,Bf)**. All images displayed are representative of at least three independent experiments. Bars correspond to 10 μm.

To obtain additional insights about the interaction and localization patterns of dichorhavirus MPs, Ns, and Ps in cell plants, we evaluated whether the characterized N-P intranuclear viroplasm complex could be rearranged from the co-expression with the cognate MP. For this purpose, initially by the expression of two protein pair combination of the MPs, Ns and Ps fused at their C-termini with GFP or RFP, we analyzed the intracellular redistributions of the proteins in *N. benthamiana* leaves. The co-expression of the N:GFP and P:RFP clearly redistributed the N protein from the cytoplasm to the nucleus for both viruses (Figures 5Aa,Ba). When we evaluated the localization of the P:RFP in the presence of MP:eGFP, we did not detect any disturbance in the localization of each protein of both viruses. Part of the MP was maintained into the nucleus, but a larger part of it localized at the cell periphery, whereas the P protein remained predominantly into the nucleus (Figures 5Ab,Bb), which is in disagreement with what was observed in BiFC analysis. On the other hand, when the MP:eGFP of CiLV-N was co-expressed with its cognate N protein tagged with the HA epitope (N:HA), the punctate MP structures visualized in some cell nuclei from leaves expressing only the MP:eGFP construct (see white arrow in Figures 3Aa, 5Acii) were no longer visualized (Figure 5Ac), suggesting that the N could aid the release of the MP from the nucleus to the cytoplasm. There were no changes in the localization of the CiLV-N N protein when co-expressed with its cognate MP:HA (compare Figure 5Ad with Figure 3Bb), which remained in the nucleus and in the cytoplasm. For the OFV-citrus, the co-expression of the MP:eGFP with the cognate N:HA did not affect MP distribution, which remained in both the nucleus and the cytoplasm (Figure 5Bc). However, the co-expression of the N:eGFP construct with the cognate MP:HA modified the nuclear localization observed for the N protein when it was expressed alone, being localized exclusively in the cytoplasm (Figure 5Bd), indicating that OFV-citrus MP redirects the N from the nucleus to the cell cytoplasm.

The observation that MP associates with N and P and can direct these proteins from the nucleus to the cell cytoplasm, suggests that the intranuclear N-P viroplasm complex could be transported out of the nucleus by the MP, in order to ensure the transport of infectious components or viral subcomplexes across the cytoplasm to neighboring cells. To address this hypothesis, we evaluated the redistribution of the intranuclear N-P complex of both OFV-citrus and CiLV-N by tagging N and P proteins with the GFP and RFP markers, respectively. The co-expression of the N and P proteins with the cognate MP:HA revealed that the N:GFP signal, seen exclusively into the nucleus in association with P (Figures 4Ae,Be, 5Aa,Ba; also see Kondo et al., 2013), was partially redirected to the cytoplasm in presence of MP, while no changes were observed for the P:RFP localization (Figures 5Ae,Be). It is noteworthy to mention that N-P colocalization signals continued to be evident in the subnuclear regions (Figures 5Ae,Be). Callose staining with aniline blue confirmed that the N:eGFP signal was redirected to the plasmodesmata at the cell periphery (Figure 5Af,Bf). These data were consistent for both CiLV-N and OFV-citrus proteins. Taken together, these findings indicate that the MP redirects the N, but not the N-P core complex, to PD at the cell periphery.

### The Dichorhavirus MPs Do Not Complement the Movement-Deficiency Phenotype of AMV, TCV, and TMV Infectious Mutants

**FIGURE 6:**
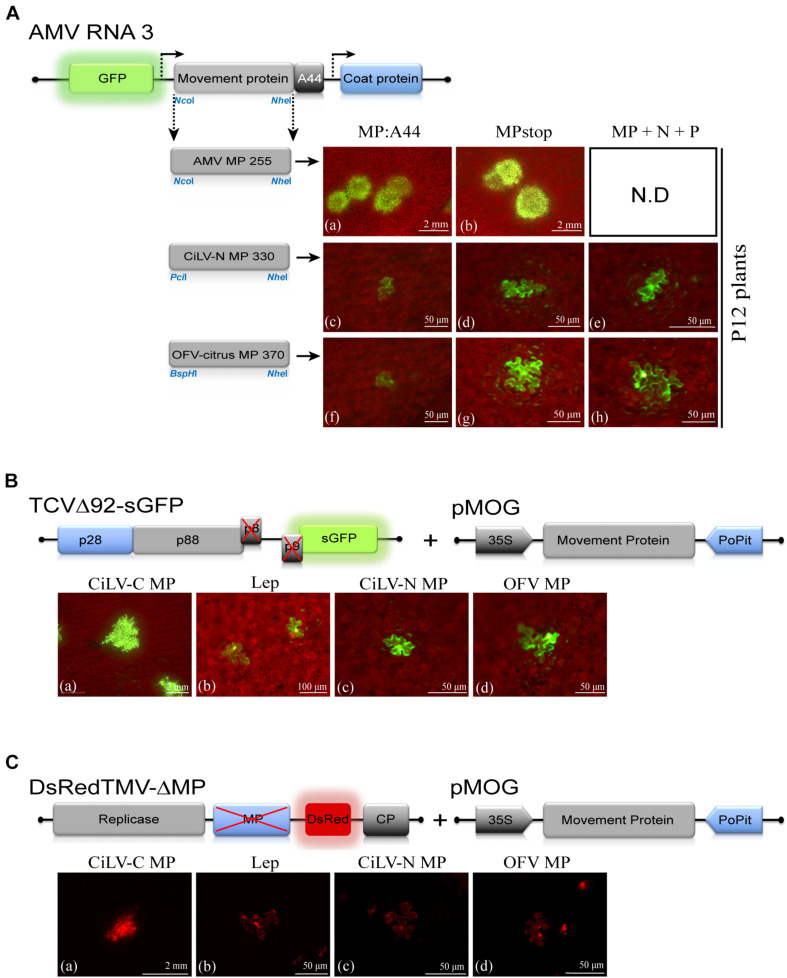
The dichorhavirus MPs do not complement the cell-to-cell movement of the alfalfa mosaic virus (AMV), turnip crinkle virus (TCV) and tobacco mosaic virus (TMV) infectious clones. **(A)** Analysis of the cell-to-cell transport of the hybrid AMV RNA 3 in which its movement protein (MP) gene was exchanged with the corresponding genes (MPs) of citrus leprosis virus N (CiLV-N) and orchid fleck virus citrus strain (OFV-citrus). Individual GFP-cell foci are observed in P12 leaves inoculated with RNA 3 transcripts from pGFP/A255/CP derivatives carrying the heterologous MPs, fused with the C-terminal 44 residues of the AMV MP (A44) (c,f) or lacking the A44 (MPstop) (d,g). Infection foci are demonstrated for the AMV MP wild-type and the mutated version expressing the N-terminal 255 residues (positive control, a,b). Representative images of all combinations from co-inoculation of AMV RNA 3 transcripts carrying the N, P, and MP dichorhavirus genes are presented (e,h). The schematic representation shows the GFP/A255/CP AMV RNA 3, in which the open reading frames, represented by large boxes, correspond to the green fluorescent protein (GFP), the movement protein (MP), and the coat protein (CP). Short box corresponds to the C-terminal 44 amino acids of the AMV MP, meanwhile arrows represent subgenomic promoters. The numbers after the viral acronym represent the total amino acid residues of the corresponding MP. The *Nco*I, *Bsp*HI, *Pci*I, and *Nhe*I restriction sites used for insertions of the MPs are indicated. White bars correspond to 50 μm–2 mm. Each infection foci image is representative of the inoculation of three leaves per plant and two plants inoculated for each chimeric AMV construct. ND, non-determined. **(B)** TCV assay based on the complementation of the movement-deficiency phenotype of a TCV MP mutant (TCVΔ92-sGFP). Three *N. benthamiana* leaves per plant were infiltrated with pMOG_800_ constructs carrying CiLV-C MP (a) (positive control) and MPs of CiLV-N (c) and OFV-citrus (d) or pMOG-Lep (b) as negative control. Infectious RNA transcript of the TCVΔ92-sGFP construct was mechanically inoculated 1 day post agroinfiltration. Cell-to-cell movement was evaluated at 3 days post inoculation. Viral movement complementation is visualized for CiLV-C MP expression (a); meanwhile, only individual foci-cells are visualized for the leaves inoculated with the constructions expressing the dichorhavirus MPs (c,d). The schematic representations show: (1) the TCV RNA, in which the open reading frames are represented by the boxes. The p28 (blue box) and p88 (gray) code for replication proteins. The p8 and p9 encode movement proteins (black box) and the green fluorescent protein (GFP) is represented in the place of the viral coat protein. An X in the p8 and p9 indicates protein deletion. (2) pMOG expression cassette used for the ectopic expression of the MPs indicated. The genes were cloned between CaMV constitutive 35S promoter (black box) and potato proteinase inhibitor terminator (PoPit, blue box). White bars correspond to 50 μm–2 mm. **(C)** TMV trans-complementation assay based on a TMV MP defective mutant expressing the red fluorescent protein (DsRed) (DsRedTMV-ΔMP). *N. benthamiana* leaves (three per plant) were infiltrated with an agrobacterial culture containing the binary pMOG constructs carrying CiLV-C MP (a), CiLV-N MP (c), OFV-citrus MP (d), or pMOG-Lep (b) as negative control. At 1 day post agroinfiltration, all infiltrated leaves were inoculated with DsRedTMV-ΔMP derived transcripts. The schematic representations show the pMOG cassette above described and the DsRedTMV-ΔMP RNA, in which the ORFs are represented by boxes corresponding to replicase (gray box), deleted MP (blue box), DsRed (red box), and the capsid protein (CP, gray box). White bars correspond to 50 μm–2 mm.

To further explore the functionality of the dichorhavirus MPs, we tentatively evaluated their intrinsic movement proprieties using two distinct approaches: *cis-* and *trans*-movement complementation analyses. For *cis*-movement complementation assay, the MP genes of CiLV-N and OFV-citrus were inserted into the AMV RNA3 infectious construction that expresses the GFP (pGFP/A255/CP), fused or not with the C-terminal 44 amino acids of the AMV MP (A44) (Sanchez-Navarro et al., 2001) by exchanging the AMV MP gene expressing the N-terminal 255 residues (A255). The presence of the A44 region at the C-terminus of the heterologous MPs allows the interaction between the heterologous MPs and the AMV coat protein. The AMV system permits the functional exchangeability of viral MPs, at least for the 30k family (Sanchez-Navarro and Bol, 2001; Sanchez-Navarro et al., 2006). *In vitro* transcripts from these heterologous constructions plus the AMV wt, were mechanically inoculated in transgenic *N. tabacum* plants constitutively expressing the P1 and P2 sub-units of the viral replicase (P12 plants), to evaluate the cell-to-cell movement. Only single fluorescent cells were observed on P12 leaves (Figures 6Ac,d,f,g) by AMV RNA3 derivatives expressing the dichorhavirus MPs, indicating the incompatibility of these proteins to complement the local AMV movement. P12 leaves inoculated with AMV transcripts expressing its MP255 and MP wt resulted in clear fluorescence infection foci at 2 days post inoculation, as expected (control, Figures 6Aa,b). Additionally, we inserted the N and P genes of both CiLV-N and OFV-citrus into the AMV cDNA3 clone, and the mix of transcripts (MP, N, and P) was co-inoculated in P12 leaves in all possible combinations: MP:A44 + N:A44 + P:A44, MP:A44 + N:A44 + Pstop, MP:A44 + Nstop + P:A44, MP:A44 + Nstop + Pstop, MPstop + N:A44 + P:A44, MPstop + N:A44 + Pstop, MPstop + Nstop + P:A44, MPstop + Nstop + Pstop. No infection foci were observed for all combinations, as shown by the representative images in Figures 6Ae,h.

To evaluate movement properties of dichorhavirus MPs based in a *trans*-movement approach, *Agrobacterium* cultures transformed with binary plasmid carrying the CiLV-N and OFV-citrus MPs were infiltrated in *N. benthamiana* leaves, and at 1 day post infiltration, they were mechanically inoculated with transcripts from the infectious TCVΔ92-sGFP mutant, which has a 92 nucleotide deletion that abolishes the expression of the TCV double gene block (p8 and p9) movement proteins (Li et al., 1998; Powers et al., 2008), exhibiting a movement-deficiency phenotype. Foci formation, not limited to three or five cells observed in the negative control (pMOG-Lep, Figure 6Bb), were visualized by expression of cilevirus p32 movement protein (positive control, Figure 6Ba). On the other hand, the dichorhavirus MPs did not trans-complement the TCVΔ92-sGFP movement, showing only single fluorescent cells (Figures 6Bc,d). Finally, we evaluated the movement properties of dichorhavirus MPs using a TMV infection construct (Canto and Palukaitis, 2002) lacking the MP gene (DsRedTMV-ΔMP). Individual fluorescent cells were visualized in *N. benthamiana* leaves inoculated with CiLV-N and OFV-citrus MPs (Figures 6Cc,d), instead of the cilevirus CiLV-C MP, which rescued the TMV movement generating large infection foci (Figure 6Ca). These results indicate that, unlike cilevirus MPs, the dichorhavirus MPs are not sufficient to rescue the cell-to-cell movement of three different viruses, which have distinct viral transport mechanisms.

### The Ectopic Expression of the Dichorhavirus MPs Does Not Induce Tubular Structures on Protoplasts

Next, we investigated whether the dichorhavirus MPs were capable of forming tubule structures on the surface of *N. benthamiana* protoplasts. The MP:eGFP of CiLV-N and OFV-citrus were transiently expressed in *N. benthamiana* leaves by agroinfiltration. The protoplasts were prepared 24 h post infiltration (hpi), and GFP signals were visualized at 16 h after protoplasts purification. None of the dichorhavirus MPs were able to generate tubular structures, showing GFP signal accumulation as punctate structures at the cell periphery without tubule polymerization (Supplementary Figures 1a,b). The cilevirus CiLV-C2 MP induced the formation of tubular structures regardless of viral infection (positive control, Supplementary Figure 1f). In the present work, we observed that the OFV-citrus MP interacts with P and N and is able to recruit the N from the N-P complex to the plasmodesmata at the cell periphery. In order to evaluate if N or P proteins of OFV-citrus could be required for the MP tubule polymerization, we prepared protoplasts from *N. benthamiana* leaves transiently co-expressing the combinations MP + N, MP + P, and MP + N + P. No tubular structures were visualized in any of the assayed protein combinations (Supplementary Figures 1c–e), further suggesting that dichorhavirus MPs are not tubule-forming proteins.

### Revealing a Broad Permissibility of *in vivo* Interactions Between Proteins of Distinct BTVs

**FIGURE 7:**
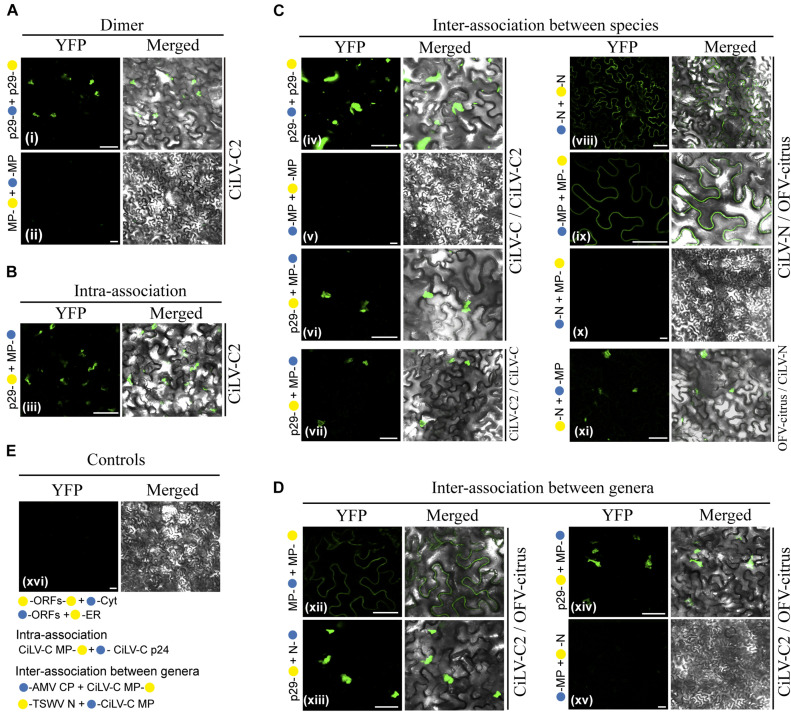
BiFC analyses of the transient expression combinations between movement proteins (MP), nucleoproteins (N), and capsid protein (p29) from distinct BTVs. Dimer **(A)**, intra **(B)**, and inter **(C,D)** interactions of the p29 and MP of the CiLV-C2, and Ns and MPs of the CiLV-N and OFV-citrus, were analyzed by BiFC. p29, Ns and MPs carrying N-terminal (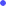) or C-terminal (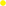) YFP fragments fused at their N- (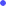/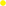-ORFs) or C-termini (ORFs-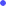/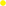) were transiently co-expressed in *N. benthamiana* leaves by agroinfiltration. Representative protein pair combinations are indicated at the left of each image; all hetero-combinations performed are shown in Table 3. All images contain two pictures corresponding to the YFP signal or merged with bright field. The YFP fluorescence reconstitution was monitored at 4 days post-infiltration using a confocal laser scanning microscope Zeiss LSM 780 model. Suggested positive interactions were visualized in (i,iii,iv,vi–viii,ix,xi–xiii,xiv). No interactions are indicated in (ii,v,x,xv,xvi). Negative controls correspond to the expression of the respective proteins in combination with Cyt or ER BiFC vectors **(E)**. Additional negative controls are presented for the intra-association: CiLV-C MP + p24 (putative matrix protein) (Leastro et al., 2018) and for the inter-association between genera: AMV CP + CiLV-C MP and TSWV N + CiLV-C MP (Leastro et al., unpublished) **(E)**. All images displayed are representative of three independent experiments. Bars correspond to 50 μm.

Mixed infection of distinct BTVs and mutual mite colonization in citrus plants has been reported for the citrus leprosis pathosystem (Roy et al., 2015a; Beltran-Beltran et al., 2020). Therefore, we analyzed by BiFC assay, whether the viral movement components, suggested here for dichorhaviruses and previously characterized for cileviruses (Leastro et al., unpublished), could perform heterologous interactions. Negative controls for each specific interaction corresponded to the expression of the viral proteins tested in combination with Cyt or ER BiFC vectors, and a representative image corresponding to all experimental negative controls is presented in Figure 7E. As additional control for intra- and inter-association analyses, we expressed the CiLV-C MP with the cognate p24 protein (putative matrix protein) or with the CP and N proteins from viruses belonging to the genera *Orthotospovirus* and *Alfamovirus* (intra-association: CiLV-C p24 vs. CiLV-C MP, inter-association: AMV CP vs. CiLV-C MP and TSWV N vs. CiLV-C MP) in all cases resulting in the absence of fluorescence (Figure 7E), as previously demonstrated (Leastro et al., 2018, unpublished). All protein pair combinations performed in these assays are shown in Table 3. The BiFC image is representative of all protein pair combinations. The homologous and heterologous interactions for the capsid protein (p29) and MP of CiLV-C were previously reported (Leastro et al., 2018). Here, we extended this understanding for CiLV-C2, another member of the genus *Cilevirus*. The BiFC assay suggests that CiLV-C2 p29 was able to form dimer structures in epidermal cells of *N. benthamiana* (Figure 7Ai). Similarly to what was observed for CiLV-C MP (Leastro et al., 2018), the CiLV-C2 MP was unable to dimerize (Figure 7Aii). The analysis of the CiLV-C2 p29-MP interaction showed a clear fluorescence reconstitution throughout the cytoplasm (Figure 7B), as recently reported for CiLV-C (Leastro et al., 2018).

Next, we tested the inter-protein association between different CL-associated cileviruses (CiLV-C vs. CiLV-C2) and dichorhaviruses (OFV-citrus vs. CiLV-N) proteins. For the cileviruses, the reconstituted fluorescence suggesting positive *in vivo* interaction was observed for the combinations: CiLV-C p29 vs. CiLV-C2 p29, CiLV-C p29 vs. CiLV-C2 MP, and CiLV-C2 p29 vs. CiLV-C MP (Figures 7Civ,vi,vii). No fluorescence was observed for the CiLV-C MP vs. CiLV-C2 MP combination (Figure 7Cv). For dichorhaviruses, fluorescence signal was observed in the CiLV-N N vs. OFV-citrus N, CiLV-N MP vs. OFV-citrus MP, and OFV-citrus N vs. CiLV-N MP combinations (Figures 7Cviii,ix,xi). On the other hand, no fluorescence signal was visualized in the CiLV-N N vs. OFV-citrus MP combination (Figure 7Cx).

**FIGURE 8:**
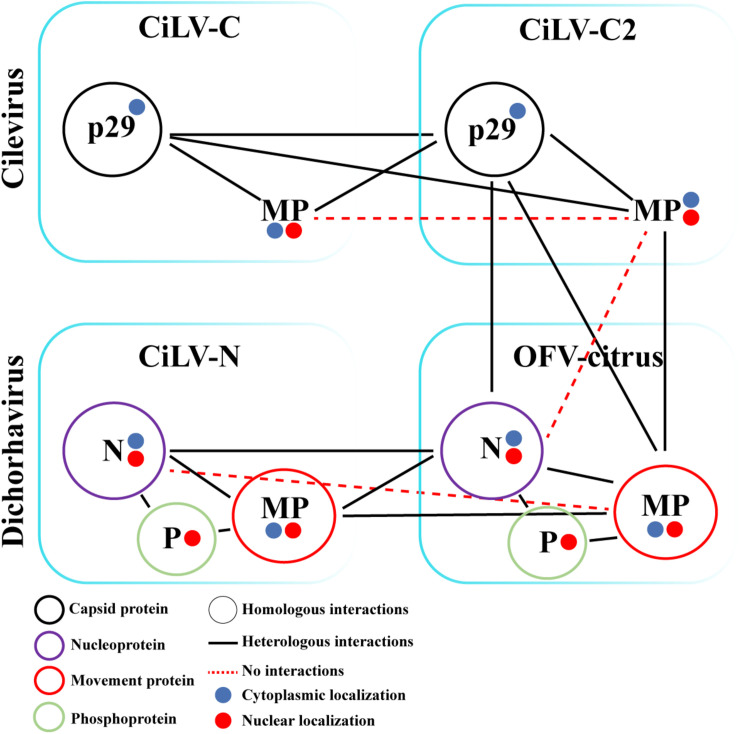
Schematic diagram of the suggested viral protein interaction and localization maps for the cileviruses (CiLV-C and CiLV-C2) and dichorhaviruses (CiLV-N and OFV-citrus) indicated proteins. The scheme indicates the suggested dimeric protein interactions as well as heterologous interactions between proteins of viral species of the same genus and between different genera associated with the citrus leprosis photosystem. Self-interactions are indicated by a color circle specific to each protein. The circles filled with blue or red indicate the cytoplasmatic or nuclear protein localization, respectively. Circularized acronyms indicate positive homologous interactions. Black lines indicate positive heterologous interactions, while red dotted lines indicate lack of positive interaction.

Finally, we analyzed the interaction (inter-association) between proteins from viruses belonging to different genera (CiLV-C2 vs. OFV-citrus). Positive fluorescence signal was visualized for CiLV-C2 MP vs. OFV-citrus MP, CiLV-C2 p29 vs. OFV-citrus N, and CiLV-C2 p29 vs. OFV-citrus MP combinations (Figures 7Dxii,xiii,xiv). No fluorescence was visualized for CiLV-C2 MP vs. OFV-citrus N combination (Figure 7Dxv). All BiFC suggesting interaction and subcelullar results obtained with the different dichorhaviruses and cileviruses proteins were used to generate a diagrammatic map of *in vivo* protein interactions and subcellular localizations for all of them (Figure 8).

## Discussion

In this report, we explored in detail different aspects related to the movement mechanism of dichorhaviruses by analyzing biological properties of the MP, N, and P proteins from viruses belonging to two different species. The analyses included membrane topology, subcellular localization, and *in vivo* protein–protein interaction between them but also with the capsid and movement proteins from distinct citrus leprosis-associated cileviruses.

The membrane fractionation analysis revealed that the MPs of two dichorhaviruses are membrane proteins strongly and physically associated to the cell membrane. This finding, together with the topology observed in the BiFC results, allows us to propose a topological model for the dichorhavirus MPs, in which the MPs are associated to membranes, but probably not integrally associated, with the full-length molecule oriented toward the cytoplasmic face of the biological membrane (see Figures 2Bxii,Cxx), and when present within the nucleus, located at the inner nuclear membrane. The capability to associate with cell membranes and topology reported here is in agreement with the pattern reported for different movement proteins of other negative and positive plant viruses belonging to the 30K superfamily, including the MPs of the evolutionary related betanucleorhabdovirus SYNV and the cytorhabdovirus tomato yellow mottle-associated virus (TYMaV) (Melcher, 2000; Martínez-Gil et al., 2009; Genovés et al., 2011; Peiro et al., 2014b; Leastro et al., 2015; Zhou et al., 2019). However, since the dichorhavirus MPs do not belong to the 30K superfamily (OFV and CiLV-N MP genes encode 41.7 and 36.6 kDa proteins, which show no homology with the 30K MPs), our data indicate that this topology is not only restricted to movement proteins belonging to that family. Moreover, it further strengthens the hypothesis that association between movement proteins with host membranes seems to be an essential factor for plant virus transport.

Both N proteins localized mainly in the cytoplasm, in the absence of other viral components. Similar subcellular localization has been reported for the OFV (orchid strain) N protein (Kondo et al., 2013). However, we observed the presence of CiLV-N and OFV-citrus N proteins in the nucleus, similarly to what was reported for the dichorhavirus coffee ringspot virus (CoRSV) (Ramalho et al., 2014), but not for the OFV orchid strain. For the latter, it was proposed that the nuclear localization of the N protein occurred through its interaction with the P protein, which possesses a nuclear localization signal (NLS) (Kondo et al., 2013). Here, we observed that, in the absence of the cognate P protein, both CiLV-N and OFV-citrus N proteins localize in the nucleus. Also, we observed that neither N proteins exhibit the NLS predicted domains (cNLS Mapper with cut-off score 5.0)^1^, as reported for the CoRSV N protein (Ramalho et al., 2014). That indicates that the nuclear localization observed could be a consequence of the protein overexpression in the agroinfiltration system. In this sense, although we cannot exclude the possibility of a nuclear active transport for both N proteins, it is important to emphasize that the nuclear pore complex (NPC) allows passive diffusion of molecules up to about 60 kDa (Wang and Brattain, 2007). The co-expression of the N and P proteins from CiLV-N and OFV-citrus resulted in a marked change of the N localization, detecting both proteins exclusively in the nucleus, in agreement with the N-P localization patterns reported for the OFV orchid strain and for nucleorhabdoviruses (Goodin et al., 2001; Tsai et al., 2005; Ghosh et al., 2008; Kondo et al., 2013). However, the P proteins were not detected in the cytoplasm upon the co-expression with the cognate N proteins, as reported for CoRSV (Ramalho et al., 2014), remaining in the nucleus at all time. This divergence could indicate that evolutionary related species belonging to the same genus may have specific models for nucleocapsid formation and/or viral replication.

In the present study we observed the presence of two different dichorhavirus MPs in the nucleus, in addition to the cell periphery. Previous studies with the CoRSV MP localized the protein predominantly at the cell periphery when ectopically expressed in plant cells (Ramalho et al., 2014). MP nuclear localization has also been reported for the lettuce necrotic yellows cytorhabdovirus (LNYV), alfalfa dwarf cytorhabdovirus (ADV), and SYNV betanucleorhabdovirus (Goodin et al., 2002, 2007; Martin et al., 2012; Bejerman et al., 2015; Zhou et al., 2019). For the LNYV MP (4b protein), its nuclear localization was supported by the presence of a predicted NLS domain, whereas no canonical NLS was predicted for ADV MP (P3 protein). Using a cNLS Mapper software, we could not identify NLS domains for either CiLV-N or OFV-citrus MPs (data not shown).

A previous study demonstrated that the CoRSV MP was unable to interact with other CoRSV-encoded proteins (Ramalho et al., 2014). Here, we have demonstrated that both CiLV-N and OFV-citrus MPs were able to interact *in vivo* with their cognate N and P proteins. The MP(P3 protein)-P interaction *in vivo* has also been reported for the cytorhabdovirus ADV (Bejerman et al., 2015). In that study, the authors also observed a partial relocalization of the MP, from the cell periphery to the nucleus, when both MP and P proteins were co-expressed. For CiLV-N, the MP-P complex obtained from BiFC analysis was distributed into the nuclei and at the cell periphery. The observation that the CiLV-N P protein is exclusively detected in the nucleus, in the absence of other viral proteins, indicates that the MP may recruit the P protein from the nucleus to the cell periphery, opening the possibility that the P protein could be implicated in the virus movement mechanism. In contrast to CiLV-N, the OFV-citrus MP-P complex was visualized exclusively into the nucleus, suggesting multiple functions for this MP. The CiLV-N N-MP complex formed in BiFC analysis was mostly distributed in the cytoplasm, where the N protein was able to relocalize the MP from the nucleus in co-expression analysis, indicating that the N protein may also assist the MP export. On the other hand, for OFV-citrus, the localization of the N-MP complex occurred mostly in the nucleus. However, co-expression analysis revealed that the N protein was redirected exclusively to the cell cytoplasm. Taken together with the localization results presented for the MP-P complex, our findings suggest that the CiLV-N MP may recruit both N and P for the movement, whereas the OFV-citrus P and N seem to recruit the MP to the nucleus, but remarkably, the opposite is also observed with the N being exported to the cytoplasm. This observation clearly demonstrates the use of different infection mechanisms for these dichorhaviruses, and more importantly, the existence of a dynamic interaction complex formed between the MP with the cognate nucleocapsid (N-P) proteins, strongly suggesting that, for dichorhaviruses, the nucleocapsid could play a role in viral movement mechanism, as observed for other plant viruses (Chapman et al., 1992; Forster et al., 1992; Nagano et al., 2001). A common property observed for both viruses was the redistribution of a portion of the N protein from the nucleus to the cytoplasm and to plasmodesma structures upon co-expression of the three MP, N, and P proteins (Figure 5). A similar feature has been recently reported for the phylogenetically related betanucleorhabdovirus SYNV, in which the viral MP (sc4) directed a portion of the N-P complex from nuclear sites of replication to the cell periphery, co-localizing partially with PD (Zhou et al., 2019). For both CiLV-N and OFV-citrus, we do not rule out the possibility that a reduced portion of the N-P complex could be redirected to the cell periphery in the presence of the MP mediated through the MP-P interaction, especially for CiLV-N. Further analyses will be addressed to confirm this hypothesis.

Our findings reveal that dichorhavirus MPs do not display cross-movement complementation with distinct _(+)_RNA viruses, suggesting either that these MPs mediate virus transport with a mechanism distinct from those viruses used in the transport complementation assay or the requirement of specific interactions and/or proteins playing a role in virus transport. For plant rhabdoviruses and others (-)ssRNA viruses, their MPs were able to rescue the cell-to-cell movement of potexvirus, tobamovirus, or alfamovirus defective-movement mutants (Huang et al., 2005; Peiro et al., 2014a; Mann et al., 2016; Leastro et al., 2017b; Zhou et al., 2019). On the other hand, defective nucleo- and cytorhabdovirus movement mutants were rescued only by their cognate MPs (Zhou et al., 2019). Thus, we speculate that the movement mechanism mediated by the dichorhavirus MP could be specific, requiring cognate viral proteins as shown for other plant rhabdoviruses. Based on our findings on redistribution and plasmodesmata localization of the nucleocapsids, it is tempting to hypothesize that the cognate N-P complex could be the specific viral accessory factor needed to ensure virus spread. In this sense, we tested the co-expression of N, P, and MP in AMV context, which was unsuccessful. This observation further suggests that dichorhavirus MPs could have a strong specificity requiring cognate factors or cis elements, in natural infection context, which cannot be reproduced in heterologous systems. The incapacity of their MPs to rescue the movement of these classical virus systems opens the possibility of the existence of a viral movement mechanism, mediated by dichorhavirus MPs, different from those known so far. An infectious clone-based study model for dichorhavirus would allow investigating in detail its movement mechanism.

The dichorhavirus MPs transiently expressed on protoplasts of *N. benthamiana* did not result in tubule formation either alone or by the co-expression with the cognate nucleocapsid proteins, suggesting that these MPs are non-tubule forming. This is in agreement with the viral non-tubule guided movement mechanism, where the MPs modify plasmodesmatal size exclusion limit without the tubule polymerization (Wolf et al., 1989). Whether dichorhavirus MPs can form tubule structures on the natural context of viral infection remains to be addressed.

Mixed infection favors genetic rearrangement of viral species and may also potentiate the process of viral infection (Roossinck, 1997; Leastro et al., 2015; Moreno and Lopez-Moya, 2020). Therefore, we explored the association between movement components of dichorhaviruses (MP, N, and P proteins) and cileviruses (MP and p29 proteins). Interactions between movement viral components have been noticed for distinct orthotospoviruses (Leastro et al., 2015), revealing broad compatibility between MPs and Ns from four tested viruses. Here, the *in vivo* BiFC analyses suggested positive interactions among almost all CP-MP, N-MP, and MP-MP combinations from different BTVs. No interactions were identified only for inter-association analyses between cilevirus MPs and between CiLV-N N and OFV MP. Although positive interactions between viral MPs with heterologous capsid proteins have also been noticed for other viral genera (Leastro et al., 2015), this aspect seems not to be a rule for plant viruses, i.e., the MPs of AMV, brome mosaic virus (BMV), cucumber mosaic virus (CMV), and papaya ringspot virus (PRSV) interact with their cognate but not with heterologous CPs (Nagano et al., 1997; Takeda et al., 2004; Sanchez-Navarro et al., 2006; Aparicio et al., 2010).

Regarding the dimer analysis of the MP (for both genera), p29 (for cilevirus), and N (for dichorhavirus) proteins, the only negative interaction noticed was for CiLV-C2 MP, which is inconsistent with the recent observation showing the capacity of this protein to polymerize, forming tubular structures on the protoplasts surface (Leastro et al., unpublished). We speculate that dimerization of this MP could be incompatible with the fusion of the YFP fragment or with the right orientation of the two YFP fragments.

The BiFC analyses using proteins from distinct BTV (CiLV-C2 vs. OFV-citrus), also suggested a broad compatibility interaction among MPs, CPs, and Ns proteins. The permissibility reported here agrees with the report of mutual mite colonization and mixed CiLV-C2 and OFV-citrus infection in the same lesion (Roy et al., 2015a); although, in nature, the chance of CL-associated viruses to interact with each other can be considered very low, since they do not spread systemically in their hosts, remaining localized in the lesions they induce (Freitas-Astua et al., 2018). Among all the *in vivo* protein–protein interactions evaluated for CiLV-C2 and OFV-citrus, the only negative interaction was observed between CiLV-C2 MP and OFV-citrus N, although we have observed interaction between OFV-citrus MP and CiLV-C2 CP, suggesting that the dichorhavirus MP could assist the transport of both cognate nucleocapsid and heterologous capsid proteins in the mixed infection process. In this sense, we have observed that the dichorhavirus MP redirects the cognate N from the nucleus to the plasmodesma at the cell periphery. However, to determine whether this capability could be extended for the transport of the capsid protein of a non-genetically related virus, further tests are required. Considering the mixed infection between species in the CL pathosystem, heterologous interactions between viral proteins of distinct species could represent a potential to generate synergism. Further analysis will be addressed to confirm the putative synergism, based on heterologous protein–protein interactions, in mixed infections of BTV.

## Data Availability Statement

All datasets generated for this study are included in the article/Supplementary Material, further inquiries can be directed to the corresponding authors.

## Author Contributions

ML and JS-N conceived and designed the experiments and analyzed and interpreted the data. ML performed the experiments and wrote the original draft. ML, JF-A, EK, VP, and JS-N contributed with reagents, materials, and tools, revised and edited the manuscript. All authors contributed to the article and approved the submitted version.

## Conflict of Interest

The authors declare that the research was conducted in the absence of any commercial or financial relationships that could be construed as a potential conflict of interest.

## Footnotes

1 http://nls-mapper.iab.keio.ac.jp/cgi-bin/NLS_Mapper_form.cgi

## Supplementary Material

Supplementary Figure 1 The MPs of dichorhaviruses do not induce tubular structures on protoplast. Analysis of tubule formation of *N. benthamiana* protoplasts transiently expressing the MP genes of CiLV-N **(a)**, OFV-citrus **(b)**, and CiLV-C2 (**f**, positive control) fused at their C-termini with eGFP (). Three infiltrated leaves per construct were used for protoplasts isolation. Protoplasts were purified after one day post-infiltration and the fluorescence GFP signal was captured 16 h post-protoplasts purification with a Zeiss LSM 780 confocal laser-scanning microscope. The green (GFP), transmitted light (TL) channels and merged images are shown. Each image-frame expressing GFP represents the visualization of several protoplasts (about 15 to 20) per assay for each MP protein analyzed. Protoplast preparations from co-infiltration of the OFV-citrusMP:eGFP with cognates N and P carrying a C-terminal HA tag is presented. Tubule formation is observed from expression of the CiLV-C2 MP **(f)**. GFP signal in punctate structures on surface of protoplast is visualized for all other **(a–e)** dichorhaviruses proteins expressed **(c)**. Red bars correspond to 10 μm.
