# Supplementary figures and images for: Dichorhaviruses Movement Protein and Nucleoprotein Form a Protein Complex That May Be Required for Virus Spread and Interacts in vivo With Viral Movement-Related Cilevirus Proteins
Source: Front Microbiol. 2020 Nov 4;11:571807. doi: 10.3389/fmicb.2020.571807 (PMC7672204; doi:10.3389/fmicb.2020.571807)

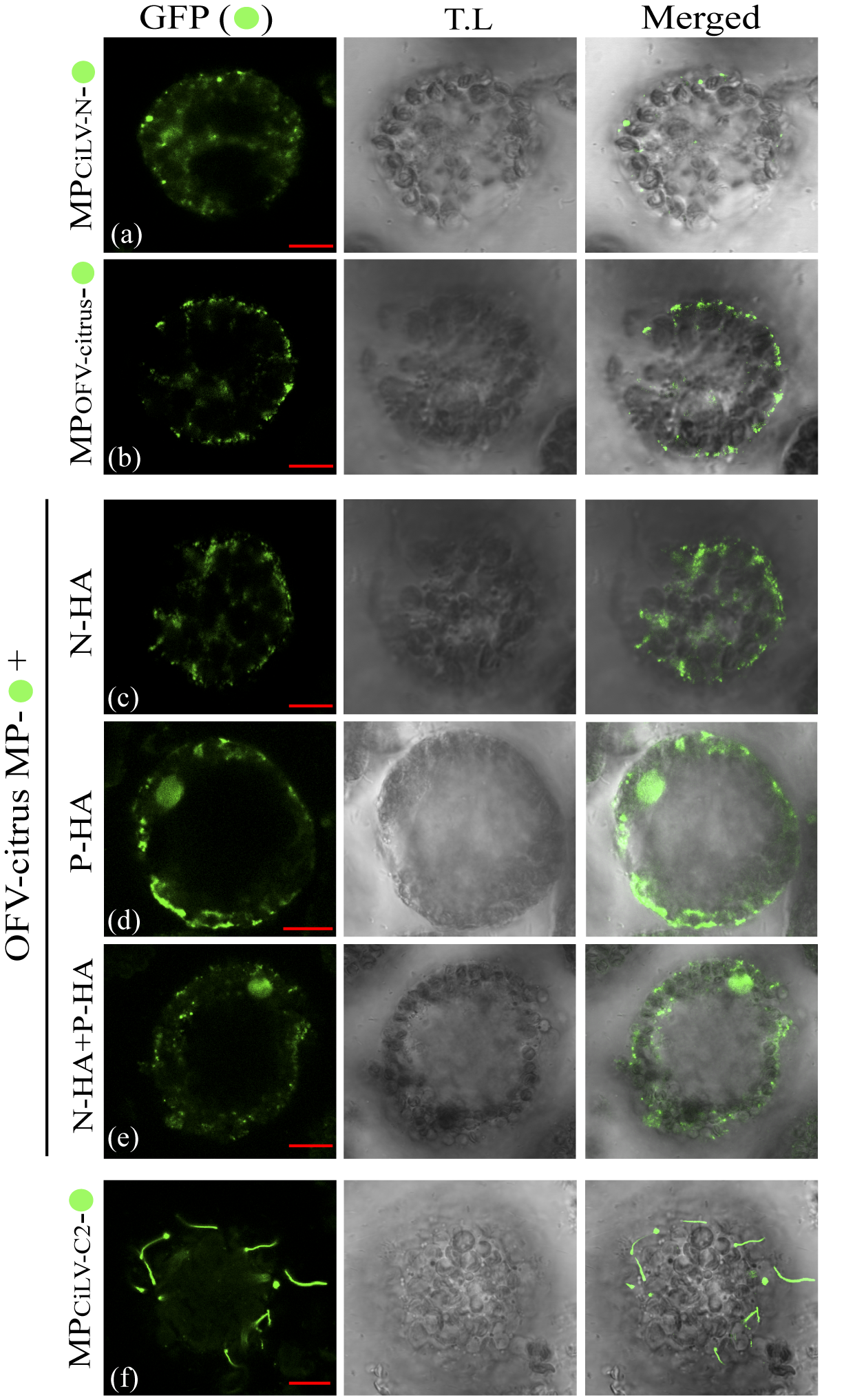

Supplement: Supplementary Figure 1 — The MPs of dichorhaviruses do not induce tubular structures on protoplast. Analysis of tubule formation of N. benthamiana protoplasts transiently expressing the MP genes of CiLV-N (a), OFV-citrus (b), and CiLV-C2 (f, positive control) fused at their C-termini with eGFP (). Three infiltrated leaves per construct were used for protoplasts isolation. Protoplasts were purified after one day post-infiltration and the fluorescence GFP signal was captured 16 h post-protoplasts purification with a Zeiss LSM 780 confocal laser-scanning microscope. The green (GFP), transmitted light (TL) channels and merged images are shown. Each image-frame expressing GFP represents the visualization of several protoplasts (about 15 to 20) per assay for each MP protein analyzed. Protoplast preparations from co-infiltration of the OFV-citrusMP:eGFP with cognates N and P carrying a C-terminal HA tag is presented. Tubule formation is observed from expression of the CiLV-C2 MP (f). GFP signal in punctate structures on surface of protoplast is visualized for all other (a–e) dichorhaviruses proteins expressed (c). Red bars correspond to 10 μm. [file Image_1.TIF]
